# The clinical adoption meta-model: a temporal meta-model describing the clinical adoption of health information systems

**DOI:** 10.1186/1472-6947-14-43

**Published:** 2014-05-29

**Authors:** Morgan Price, Francis Lau

**Affiliations:** 1 School of Health Information Science, University of Victoria, Victoria, British Columbia, Canada; 2 Department of Family Practice, Faculty of Medicine, University of British Columbia, Vancouver, British Columbia, Canada

**Keywords:** Health information systems, Adoption model

## Abstract

Health information systems (HISs) hold the promise to transform health care; however, their adoption is challenged. We have developed the Clinical Adoption Meta-Model (CAMM) to help describe processes and possible challenges with clinical adoption. The CAMM, developed through an action research study to evaluate a provincial HIS, is a temporal model with four dimensions: availability, use, behaviour changes, and outcome changes. Seven CAMM archetypes are described, illustrating classic trajectories of adoption of HISs over time. Each archetype includes an example from the literature. The CAMM and its archetypes can support HIS implementers, evaluators, learners, and researchers.

## Background

Health information systems (HISs) have been described as one of the key tools to transform and improve quality of our healthcare systems [1,2]. However, the promise of these transformative tools has not been consistently seen [3-5] and meaningful adoption in many jurisdictions remains low [6,7]. The deployment of HISs has met with a wide variability in outcomes from benchmark successes that lead to transformations in care [8] to never being deployed in a clinical setting. Adoption of HISs has been a significant and increasing concern in healthcare [9] and an important problem to be addressed [10]. Adoption needs to be better described and understood with approaches that are accessible to people planning and implementing these systems.

In this paper we present the Clinical Adoption Meta-Model (CAMM). The CAMM has been developed to describe HIS adoption over time. First, we provide background on adoption models. Then we present the CAMM, followed by seven CAMM archetypes that illustrate classic adoption patterns. We finish this paper with a discussion on how the CAMM and its archetypes can support implementers, evaluators, learners, and researchers.

### Adoption models

Adoption is the process that “involves the multitude of activities, decisions, and evaluations that encompass the broad effort to successfully integrate an innovation into the functional structure of a formal organization” [11] (p. 5). An adoption model provides a simplified and limited explanation of the complex process of integration over time. For information systems, this involves the complex sociotechnical aspects that occur over time from initial deployment through to integration into practice [12]. For this paper, we will use the term adoption model to describe models related to the activities of integration into practice post deployment. Adoption models, while they can be quite different, should have a number of common features to be considered an adoption model. These are: (a) they describe a number of dimensions related to adoption; (b) they are designed for a specific audience; (c) they allow for variability in assessment [13].

Outside of healthcare, there are many general adoption models, such as: Technology Acceptance Model(TAM) [14,15] and the TAM 2 [16], Unified Theory of Acceptance and Use of Technology (UTAUT) [17], IS Success model [12], Task-Technology-Fit Model [18], and the Concerns Based Adoption Model (CBAM) [11]. Diffusion of innovation [19,20] describes the spread (adoption) of an innovation (idea, process, technology) through channels within a social system. Adoption models such as the Capability Maturity Model Integration (CMMI) focus on maturity of the processes of the team managing the development [21,22]. Models like the CMMI are important for HIS development/configuration, but for clinical adoption, we are more interested in how the tools integrate into clinical practice.

### Adoption models in healthcare

Several existing adoption models have been applied to healthcare and to healthcare technology. An extensive review of diffusion of innovation in healthcare [23] recommends we seek to better understand why innovations are rejected (discontinued) once adopted. The CBAM has been applied to telemedicine [24]. TAM has been used in over 20 studies in healthcare to inform use acceptance and adoption [25]. TAM2 has also been applied [26].

Adoption models have been developed specific to healthcare. The Fit between Individuals, Task and Technology (FITT) framework highlights that adoption depends on alignment of three factors: technology, individual and task [9]. HOT-fit was used to understand critical adoption factors for HISs [27]. The Clinical Adoption Framework [28] contextualizes the IS Success Model [12] into healthcare and extends it by providing meso and macro level factors that can influence the adoption of clinical information systems. The Design-Reality Gap Model from Heeks [10] outlines seven dimensions from information to management systems and structures related to HIS failure.

Adoption models have been developed for specific domains within healthcare. HIMSS Analytics provides three EMR adoption models (EMRAM), one each for US hospital based HISs, Canadian hospital HISs, and for US Ambulatory EMRs [29,30]. Each of the three EMRAMs provide an eight-point (0–7) scale of adoption of features of the HIS. Diagnostic imaging have models to describe capability for collaborative jurisdictional infrastructure maturity [31]. The PACS maturity model [32,33] describes the process maturity of hospital based PACS systems in terms of functionality and integration into practice workflow. The EMR (Electronic Medical Records) Adoption Model [34] provides an adoption assessment tool that breaks down office-based EMR adoption into 10 functional areas.

What appears missing from these models is a contextualized adoption model that is (a) generic to health, but (b) is sufficiently contextualized as to be accessible to key stakeholder audiences such as clinicians and administrators that (c) ties together HIS adoption and clinical benefit over time to guide expectations of adoption over time. Next, we present our Clinical Adoption Meta-Model (CAMM) that attempts to do that.

### Clinical Adoption Meta-Model (CAMM)

#### CAMM development and evaluation

**Figure 1:**
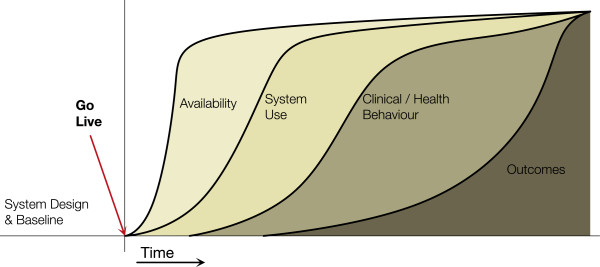
**The Clinical Adoption Meta-Model with its four dimensions.** (see http://ehealth.uvic.ca/methodology/models/CMM.php).

The CAMM (Figure 1) was developed at the eHealth Observatory (ehealth.uvic.ca) to address needs in an ongoing action research and evaluation project that is assisting the benefits evaluation of the deployment of a provincial HIS in Canada. The CAMM was developed to address the need to situate the evaluation throughout the adoption process, providing early and ongoing evaluation. The action research project has moved through three phases: assessing the initial evaluation plan, provided a revised evaluation approach, and implementing the revised evaluation while the HIS was adopted. The project is currently in the implementation phase.

Through multiple stakeholder meetings during the first two phases, it became clear that a common understanding of HIS adoption over time was needed. Several of the existing models that were considered did not resonate with stakeholders. There were three key gaps: 1. Some models did not fit the deployment/time focus of the stakeholders. 2. They were too complex and thus not engaging. 3. They were too general and did not help focus the discussion of evaluation. The primary need of stakeholders was to have an approach to understanding how to assess their HIS program benefits over time, so that realistic expectations could be set while still ensuring metrics could be measured in a timely manner to show progress. The CAMM grew out of reflections by the research team between iterations in this ongoing action research study.

Evaluation of CAMM has been primarily through the action research project. The CAMM was used to assess the initial evaluation plan, highlighting gaps to stakeholders. It was then subsequently used to co-develop a new evaluation plan that addressed the life cycle of adoption of a provincial HIS. It is now being used through phases of adoption evaluation.

The CAMM has since been used as a discussion and planning tool used with IT leadership, clinicians, IT implementers outside the above mentioned project with other stakeholders who are planning implementations of HIS and also with students who are learning about HIS adoption.

CAMM is a time dependent meta-model, with four dimensions related to post-deployment adoption: availability, use, clinical behaviour, and clinical outcomes. The dimensions are themselves dependent on each other: HIS availability is required before use can occur, use is needed to attribute changes in clinical/health behaviours to the HIS, and behavior changes can result in clinical outcomes change. Each dimension in the CAMM can have one or more aspects, which are dependent on the scope of the HIS implementation being considered. The dimensions and their aspects are described in more detail below. The Y-axis measures indicators for each dimension, the specifics of which necessarily vary from HIS to HIS.

The CAMM is designed to be generic such that it could be applied to a hospital information system deployment, an office based EMR, a personal health record, or a health app on smart phones. It could support thinking around deployment of a full HIS, a specific feature, to a new group of users, or it could be used to consider regional or jurisdictional adoption.

#### Dimension 1. availability

The first dimension, availability*,* is defined as ability for the end users to interact with an HIS. Availability includes three aspects: user access; system availability; and availability of content in the HIS. User access considers the ability for intended users to access the system (i.e. they have accounts, have been trained). System availability considers how available the HIS is to the intended users (e.g. uptime, physical availability of terminals, remote login capabilities, mobile applications). Content availability considers depth and breadth of content that is available in the HIS. This will vary, depending on the HIS, such as various types of patient data or knowledge content.

#### Dimension 2. system use

The CAMM’s second dimension, use, is defined as the interactions with the HIS by intended end-users. There are two aspects to use: use of the system and user experience of the system. Use refers to actual use of the application, such as: logins, time using the system, features used. The user experience aspect is a subjective experience of use that combines the user’s internal state, the information system (in this case the HIS), and the context in which the interactions occur [35]. User experience is broader than user satisfaction or usability. Demonstrated use of the HIS is required in order to make any assumptions correlating the HIS to changes in clinical behaviours or clinical outcomes.

#### Dimension 3. clinical/health behaviour

The CAMM’s third dimension, clinical/health behaviour, is defined as meaningful adaptation of clinical workflows or health behaviours that are facilitated by the HIS. These can align with clinical/business goals or be unintended changes in behaviour of adopting the HIS. Clinical behaviours differ from system use in that the users are incorporating information and functions provided by the information system into activities not directly related to the use of the system. Aspects of clinical behaviour change include general capacity and specific clinical activity changes. Capacity describes changes in the overall ability of the healthcare organization, which can be impacted positively or negatively by use of the HIS. Clinical activities are those specific behaviours that are impacted by HIS features. Clinical/health behaviours could be seen in providers and patients.

#### Dimension 4. clinical outcomes

The final dimension, clinical outcomes, is defined as the impacts attributable to the adoption of the HIS. There are five aspects to clinical outcomes that can be considered: patient level outcomes, provider level outcomes, organization level outcomes, population level outcomes, and cost outcomes. Measures of outcomes can be early or late. Early or surrogate outcomes are often needed in cases where it is not possible to wait for final or later outcomes.

Combined, these four dimensions make up an adoption trajectory from HIS deployment to changes in clinical outcomes. Time, which links all dimensions together in this model, could be considered a fifth dimension. The dimensions and aspects are summarized in Table 1 along with example indicators. Next, we present seven *CAMM archetypes* that show different adoption trajectories.

**Table 1:** Dimensions and Aspects of the CAMM, each dimension can have several more detailed aspects that can be considered

**Dimension**	**Aspects**	**Example indicators**
**1 – Availability**	- System availability	- Percentage of uptime of an EMR
	- User access	- Number of users with accounts for a Personal Health Record.
	- Content availability	- Months of dispensing data for a dispensing record.
**2 – System use**	- Use	- Number of log ins/user/month
	- User experience	- Survey of user experience.
**3 – Clinical behaviour**	- General capacity	- Number of patients seen per day in office.
	- Specific behaviours	- Rates of blood pressure screening
**4 – Clinical outcomes**	- Patient outcomes	- Change in blood pressure
	- Provider outcomes	
	- Organizational outcomes	- Nosocomial infection rates
	- Population outcomes	- Rates of obesity
	- Cost outcomes	- Changes in relative cost curves for an organization

Indicators can be developed based on the aspects for each dimension, as relevant to the particular HIS and the context of the adoption.

### CAMM adoption archetypes

The following seven archetypes were developed to describe typical adoption trajectories for clinical information systems. Not all of these archetypes lead to success. Many HIS implementations fail in some way [36] and thus, the archetypes capture a range of results from successful adoptions to aborted adoptions. For each archetype, we describe the adoption pattern, highlight some potential implications, and provide an exemplar study from the literature that illustrates the archetype. The example studies were selected from the corpus of eHealth studies from two recent systematic reviews [4,5]. Archetypes may be useful in teaching on HIS adoption, when considering success or challenges of a specific HIS adoption, or when planning implementation and evaluations with stakeholders.

The prototypical adoption archetypes are:

1. No Deployment.

2. Low Adoption.

3. Adoption without Benefit (behaviour and outcome).

4. Behaviour Change without Outcome Benefit.

5. Adoption with Outcome Benefits.

6. Adoption with Harm.

7. Benefit without Use.

#### No deployment archetype

This archetype describes an HIS that is not released to its users. There could be many reasons that this occurs, such as: the HIS is not completed, a flaw prevents deployment, or strategic changes prevent implementation. In this model, all four curves are flat (not shown).

The decision support application for primary care, EGADSS (Evidence-based Guidelines And Decision Support System) [37-39], is an example of this archetype. It was developed as an EMR component that connected to an EMR to provide CDSS reminders. However, it was not deployed to end users due to changes in funding and lack of adoption of the standards used in the interface between the EGADSS product and the primary care EMRs.

#### Low adoption archetype

**Figure 2:**
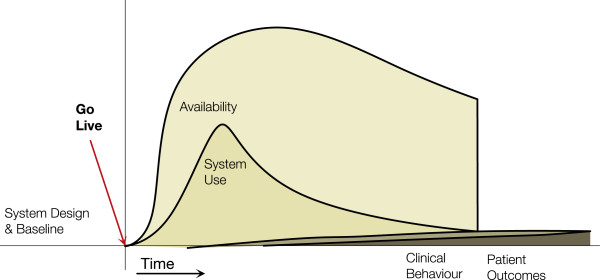
**Low Adoption Archetype.** Availability increases and there may be a surge of use, but use tapers off and the project is halted.

In this archetype (Figure 2) we see availability of the system increase but use is not sustained. No behaviour changes or outcome benefits are seen that can be attributed to the HIS. This can happen where HIS use is voluntary and it does not fit the clinical environment. Users may trial it after being given access (thus the rise in use in the diagram), but when value is not perceived or it is too cumbersome to use, then use diminishes or stops.

An example is the 2-year pre/post cluster randomized controlled trial by Eccles et al. [40] to evaluate the use of computerized guidelines in managing adult patients with asthma and angina in primary care. Sixty general practices with EMRs in northeast England took part in the study where half adopted a guideline decision support software module. At the end of the 1-year intervention, the authors found the module had no significant effect on consultation rates; process of care measures or patient reported outcomes. On closer examination, the system usage log files revealed little to no usage of the module. The authors concluded that the low adoption was likely due to the guidelines not being consistent with the practice norms, the software not integrated into the clinical workflow, and insufficient user training. Thus lack of use precluded it having impacts on behaviour or outcomes.

#### Adoption without benefits archetype

**Figure 3:**
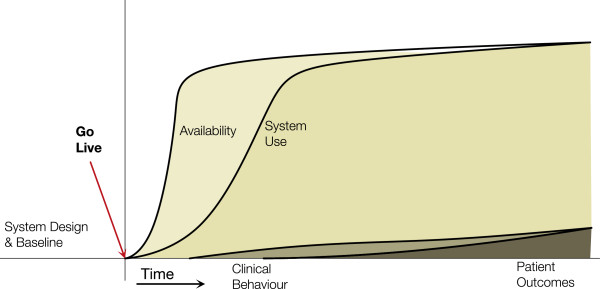
**Adoption without benefits archetype.** Availability and use increase has planned, but the corresponding behaviour changes and outcomes are not seen.

In this archetype (Figure 3) we see an HIS that is available and is used (e.g. clinicians, patients) but the expected benefits, behaviours and outcomes, do not occur. This can be seen when use is mandated or embedded in a current workflow. Benefits may not occur for several reasons, such as: an existing process is simply replicated, changes are not evidence-based, end-users chose not to incorporate the features, they are constrained by capacity, good practice was already present (i.e. a ceiling effect is reached for the indicators), or the duration of evaluation is not sufficient to see changes.

An example is the 1-year randomized controlled trial by Tierney et al. [41] on the effects of computerized guidelines in managing heart disease. 706 outpatients with heart disease were followed for 1 year where they made 3,419 primary care visits and were eligible for 2,609 cardiac care suggestions. During the study, 201 physicians in the intervention group had cardiac care suggestions displayed based on data they entered into the EMR in the encounter. The study showed no measurable effects on physicians’ adherence to care suggestions, health care utilization and costs, and patients’ medication adherence, satisfaction with care, and quality of life. The authors reported that physicians felt the guidelines provided useful information, but were constraining their management of individual patients. Although the HIS was used, clinical behaviours or outcomes were not influenced.

#### Behaviour change without outcome benefits archetype

**Figure 4:**
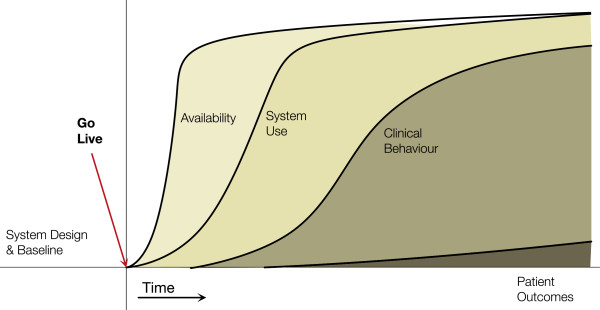
**Behaviour change without outcome benefits archetype.** Behaviours change, likely through availability and use of the HIS, but clinical outcomes are not seen.

This archetype (Figure 4) highlights a key challenge of HIS implementations: even though the system is being used and an intended behaviour change occurs, expected outcomes are not realized. This can occur for a number of reasons: outcomes are already good (i.e. a ceiling has been reached); a longer time is required to see the clinical outcome; the behaviours supported are not linked to the outcome. The last one suggests that the intervention enabled by the HIS is not evidence-based or not appropriate for the setting in which it was deployed.

An example is the interrupted time series study by van Doormaal et al. [42] on the effect of electronic prescribing on medication errors and preventable adverse drug events in two medical wards at a university medical centre in the Netherlands. Medication orders, errors, and adverse events were tracked for 5 months each before and after the implementation of a computerized provider order entry system with decision support for drug-drug interactions, overdosing, and allergy alerts. The pre/post percentages of medication errors and adverse drug events from 1,195 in patients were compared for differences. The system led to a significant reduction in medication orders with one or more medication errors (behaviour change) but not in preventable adverse events (no outcome change). The authors concluded the system lacked advanced decision support rules in therapeutic error detection and a relevant hospital formulary database in order to reduce adverse events.

#### Adoption with benefit archetype

This archetype (see Figure 1) is the archetype that HIS implementers expect to see. There is a correlation between HIS availability, use, behaviour, and the expected outcomes. See the initial description of the CAMM above.

An example is the 5-year retrospective time series study by Cook et al. [43] on the effect of an electronic medical record on antimicrobial use and nosocomial infections at a tertiary care hospital in the United States. In this study, the pattern of antimicrobial drug use, number of medical charts reviewed, number of antimicrobial recommendations made and accepted, and rates of Clostridium difficile and methicillin-resistant Staphylococcus aureus infections were assessed. Data was reviewed for 10 quarters before and 10 quarters after implementing the electronic medical record. Results were compared for differences. The study found significant increase in the number of charts reviewed (use), antimicrobial recommendations made and accepted after implementing the system (behaviour). There were decreased use of 41 commonly used antibacterial agents and reduced rates of nosocomial infections during the study period (early outcome). This retrospective study showed a correlation between HIS feature in use, clinical behaviour, and outcomes.

#### Benefit without use archetype

**Figure 5:**
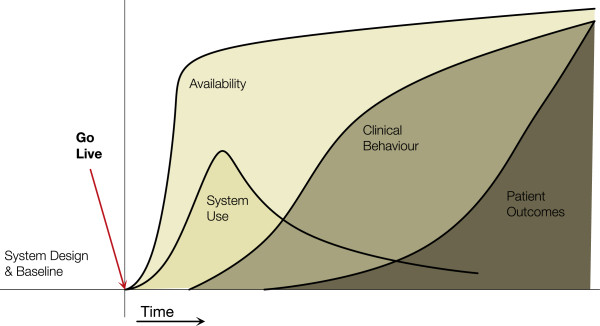
**Benefit without use archetype.** Adoption does not occur as expected, but still a clinical benefit is measured. This should not be attributed to the HIS, but a confounder.

In this archetype (Figure 5), we see the expected improvements in behaviour and outcomes; however, these occur without use of the HIS (or the specific feature of the HIS). In this archetype, one or more confounding interventions have occurred to achieve the expected improvements. In complex environments (e.g. healthcare) multiple interventions can often be occurring simultaneously, making the assessment of actual impact of HISs challenging.

Finding a published example of this archetype proved challenging. Perhaps this is because HIS evaluations are often focused on the intervention. Several studies in our corpus may fit this archetype, but they did not explicitly measure use. This was also seen in Dorr’s systematic review of HIS use in improving chronic disease management: “lack of use of otherwise successful components in standard EHR implementations should be quantified and barriers for use identified” [44] (p.162).

#### Adoption with harm archetype

**Figure 6:**
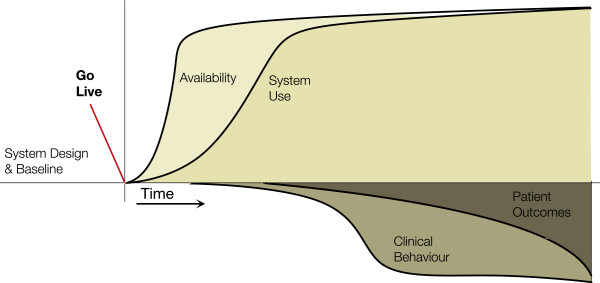
**Adoption with harm archetype.** Unintended consequence(s) occur after the deployment and adoption of the HIS resulting in a measured harm.

This archetype (Figure 6) illustrates that use of an HIS can lead to negative behaviours and/or outcomes. This is the opposite of the intention of HIS adoption and is one to avoid. Harms should be looked for during adoption of an HIS as they can occur in unexpected ways. These are unintended consequences of adoption. The expected outcome may/may not occur, but other aspects of care could change, resulting in unexpected harm.

An example is the comparison of diabetes care in primary care practices by Crosson et al. [45] where a cross sectional analysis of 50 primary care practices was completed examining quality of diabetes care through a random sampled chart audit against process guideline and outcome targets. 37 practices were paper based and 13 used EMRs. Multivariate analysis showed that EMR using practices were less likely to be following diabetes care process guidelines (clinical behaviour) and had worse intermediate outcomes (A1c, cholesterol, and blood pressure outcomes). Unfortunately, the authors did not collect duration of EMR use or any other characteristics of EMR adoption (e.g. type of EMR, features available, features used) that could be used to better explain the findings.

### Discussion

Adoption rates for HISs are variable and there are range of reasons for low adoption rates [46,47]. The CAMM was developed from a need to provide a focused description of how HIS benefits could be achieved over time (or not) and to fill a perceived gap in the literature. Highlighted in Table 2, the CAMM differs from many generic adoption models, such as the diffusion of innovation, TAM, and UTAUT, as it is contextualized to the clinical domain. And, unlike several clinical adoption models, such as the PACS maturity model [32,33], the HIMSS EMRAMs [29,30], or EMR Adoption Model [34], the CAMM is less focused on purely feature adoption and is also is generic enough to be applied to a range of clinical adoption contexts and clinical information systems from personal health records to hospital systems. CAMM is explicitly focused on the measurable outcomes that are linked to integration of IT into clinical practice over time, unlike the FITT [9] or Design-Reality Gap model [10]. Thus, the CAMM fills a gap in healthcare adoption models. The CAMM meets the Lahrmann criteria for an adoption model [13,48]: first, the CAMM has multiple dimensions (they describe clinical adoption over time); second, the CAMM allows for variability in assessment (methods are not specified); third, the CAMM is developed for specific audiences. Several audiences were considered as the CAMM was developed: implementers, evaluators, learners, and researchers. How the CAMM can support each of these audiences is described below.

**Table 2:** Comparison of the CAMM with other adoption models, CAMM is most focused on linking early adoption (e.g. availability and use) to later adoption benefits in the context of healthcare

**Model**	**Time dependent**	**Contextualized to health**	**Applicable to a range of HISs**	**Links adoption with clinical benefit**
TAM	No	No	Yes	No
UTAUT	No	No	Yes	No
Diffusion of innovation	Yes, explicit	No	Yes	No, on diffusion
IS success	Implicit	Is through Canada Health Infoway BE Framework and Clinical Adoption Framework	Yes	Yes, on net benefits.
FITT	No	No	Yes	No, focused on fit of individuals, tasks and IT.
HIMSS EMRAMs	Implicit (scores should increase over time)	Yes, to EMRs	Several EMRAMs for different EMRs	Focused on adoption of EMR features.
PACS maturity model	Implicit	Yes, to PACS	No, PACS specific	Focused on PACS features and integration, not benefit
EMR adoption model	No	Yes, to EMRs	No, office EMR only.	No, focused on EMR use.
Design-reality gap model	No	Yes, developed in hospital	Yes	No, focused on gaps.
The CAMM	Yes, explicit	Yes	Yes	Yes

#### Implementers

The CAMM can support implementers as they deploy an HIS (or HIS feature). In planning, they can use the CAMM to link goals (i.e. clinical outcomes) backward to expected behaviour changes, use, and availability, thus helping to describe the functionality and deployment needs through a causal chain. For example, if a diabetes chronic disease management tool is being deployed, the desired outcomes may be related to mortality and hospital use and, more proximally, A1c and other surrogate markers. The implementation team could consider how to structure training related to the tool use and to focus on behaviours related to reaching those goals (e.g. patient recalls). Availability could be structured to ensure the proper clinicians have access to the tool at appropriate locations and use could be promoted with those key groups. The CAMM can also help set the expectations of management, describing the processes required to reach the desired outcomes and why these are not seen immediately.

#### Evaluators

If the CAMM is used as a model to support HIS adoption evaluation, metrics assessing each of the four dimensions should be considered and be conceptually linked to each other. The further from go live, the harder it is to attribute observed changes directly to the HIS. Thus a tight linkage and clear temporal relationships between availability, use, behaviour and outcomes will strengthen attribution. Also, the CAMM highlights the importance of evaluating all four dimensions to ensure “Benefit without Use” is not occurring. CAMM does not specify evaluation methods; however, the CAMM can help theoretically link multi-methods across dimensions. The CAMM was especially useful in the action research study in which it was developed to both set expectations and to determine appropriate dimensions to measure that would align with stakeholder needs. Specifically, the need to provide progress reports on the program at specific times. The CAMM helped facilitate a discussion on what would be reasonable to evaluate for each reporting period. Through using the CAMM, evolving metric sets were considered that evaluated access, use, behaviour and ultimately outcomes over time.

#### Learners

Health informatics students and clinicians learning about HIS adoption can apply the CAMM as a framework for understanding adoption and impact of HISs over time. The CAMM and archetypes can be used with learners as a tool to describe, compare, and discuss implementation case studies.

#### Researchers

The CAMM provides a framework to consider the stages of integration into practice that could be helpful in aiding researchers design studies on adoption. It can help researchers better understand challenged implementations. We have provided some examples in this paper. The CAMM provides a common model to compare adoption of HISs (or features) for meta-analyses.

#### Limitations and future work

There are several areas of future work for CAMM. The CAMM was developed through an action research project and, while it has been informally used in a number of settings since, a more formal evaluation is needed. Specific timeframe for adoption is not specified in the generic CAMM; however, it would be valuable to understand the expected timing of each curve for various kinds of HIS adoptions: office based EMR, hospital information systems, and personal health records. Better understanding the frequency of “Benefit without Use” is needed [44]. There is a need to consider HISs as complex systems [9,49] and the CAMM can support the assessment by providing a time based model against which one can assess complex interventions. CAMM intentionally focused on the direct aspects of adoption (i.e. the four dimensions). Indirect aspects of adoption were explicitly out of scope as this model was developed. Indirect aspects, however, will impact adoption in a complex system. This is a limitation and area for future development. The CAMM, while contextualized to healthcare (e.g. clinical outcomes), could have applications in other domains (e.g. in education one could consider learning behaviours and learning outcomes for learning tools).

### Conclusion

The CAMM is presented to address a gap in the literature that was highlighted through an action research and evaluation project. It is an adoption meta-model that is focused on the temporal dimension of clinical adoption. It is general enough to be applied in multiple settings with multiple HISs from personal health records to hospital information systems. It is designed to be accessible by a range of audiences from implementers to learners. The seven CAMM archetypes provided are helpful in comparing varying levels of successful adoption. It highlights the importance of measuring multiple aspects of adoption over time to ensure that attribution of benefit can be realistically attributed to the use of HISs. As a new adoption meta-model, the CAMM needs additional application with further research.

## Competing interests

The authors declare that they have no competing interests.

## Authors’ contributions

MP and FL collaborated together on the development of the initial model. MP led the development of the manuscript, with FL providing the corpus of example studies for the CAMM archetypes. FL provided text on the example studies as well as detailed editorial support. Both authors read and approved the final manuscript.

## Pre-publication history

The pre-publication history for this paper can be accessed here:

http://www.biomedcentral.com/1472-6947/14/43/prepub
